# Environmental and Health Impacts of Domestic Hot Water (DHW) Boilers in Urban Areas: A Case Study from Turin, NW Italy

**DOI:** 10.3390/ijerph17020595

**Published:** 2020-01-16

**Authors:** Marco Ravina, Costanza Gamberini, Alessandro Casasso, Deborah Panepinto

**Affiliations:** Department of Environment, Land and Infrastructure Engineering (DIATI), Politecnico di Torino, corso Duca degli Abruzzi 24, 10129 Torino, Italy; marco.ravina@polito.it (M.R.); costanza.gamberini@polito.it (C.G.)

**Keywords:** domestic hot water, heat pump, environmental pollution, air dispersion modeling, environmental health

## Abstract

Domestic hot water heat pumps (DHW HPs) have spread fast in recent years in Europe and they now represent an interesting opportunity for implementing renewable energy sources in buildings with a centralized/district heating system, where DWH is generally produced by a gas boiler or an electric water heater. Replacing these appliances has several environmental benefits, including the removal of air pollution sources and the reduction of Green House Gasses (GHG) emissions. In this work, we present the techno-economic and environmental evaluation of implementing DHW HPs in Turin, where 66% of the DHW demand is covered by dedicated gas boilers. The impact of such boilers was assessed through numerical air dispersion modeling conducted with the software SPRAY (Aria Technologies, Paris, French). Results show that removing these sources would reduce yearly average concentrations of NOx up to 1.4 µg/m^3^, i.e., about 1% of monitored concentrations of NOx, with a benefit of 1.05 ÷ 15.15 M€/y of avoided health externalities. Replacing boilers with DHW HPs is always financially feasible with current incentives while, in their absence, it would be convenient for residential units with 3 cohabitants or more (51.22% of the total population), thanks to scale economies.

## 1. Introduction

Despite the undeniable improvement achieved in the last decades in developed countries, air quality is still critical in many urban areas in the world. Urban areas have a high population density and, hence, a high spatial intensity of different processes which can negatively affect air quality: car traffic, heating systems, use of solvents and other harmful substances, industrial processes, thermoelectric power stations, etc.

The improvement of air quality of cities in developed countries can generally be attributed to two main factors: the introduction of increasingly stringent standards for vehicle emissions, such as the Euro I-VI introduced since 1992 [1], and the progressive phase-out of coal and oil heating [2]. The massive introduction of gas heating has dramatically reduced particulate matter (PM) and sulfur oxides (SOx) emissions [3,4], yet nitrogen oxides (NOx) emissions are still high due to traffic and gas boilers, which have a low overall quality due to the slow rate of renewal [5,6].

The air quality issue strongly interacts with climate change, and this also holds true for heat production. For example, a massive replacement of fossil fuel boilers with wood boilers would result in a strong reduction of greenhouse gas (GHG) emissions, but recent experiences such as Thessaloniki [7,8,9] highlighted the unsustainability of the air quality impact in terms of PM and polycyclic aromatic hydrocarbons. The positive impact of heat pumps (the other main contributor to renewable heating) to climate change and air pollution strongly depends on the respective emission factors of electricity and on their energy efficiency [10,11,12,13]. With a more rigorous reasoning, such an impact depends on the marginal emission factor of electricity production needed to supply heat pumps. The marginal emission factor could greatly differ from the average one, for example, Lombardi et al. [14] highlighted that replacing gas cooking in Italy with electrical inductions would introduce strong peak loads on the national grid which, at present, are covered with carbon-intensive sources. On the other hand, heat pumps are widely acknowledged as an efficient way to store energy, e.g., absorbing production peaks from renewables [15,16,17].

Along with heat pumps, the other pillar of sustainable heat production is represented by district heating [18]. District heating provides several benefits from the energetic, climate, and air quality: they integrate different heat sources, including wood biomass, heat pumps, municipal solid waste incinerators, and industrial waste heat, in particular for low-temperature district heating (DH) networks [19], the combined heat and power production implies a better use of fossil fuels compared to the separate production of heat and electricity [20,21], and the emission factors of air pollutants such as NOx are much lower thanks to abatement measures which could not be techno-economically feasible at the scale of an individual boiler [22,23].

The benefits of district heating on local-scale air quality are well-known and demonstrated in the literature. Ravina et al. [24] recently studied the benefits of the DH network of Turin, the largest in Italy with 1.7 GW of thermal power installed and 68.9 Mm^3^ of heated volume served [25,26]. The average concentration reduction over the urban area of Turin was estimated in 0.5–8 µg/m^3^ of NOx and 0.1–0.3 µg/m^3^ of total suspended particulate (TSP). The model DIATI Dispersion and Externalities Model (DIDEM) [27] allows us to estimate the economic damage related to health effects of air pollution, or the economic benefit due to avoided emissions, based on the ExternE method. The results for the DH network of Turin are between 3.88 M€/y and 85.65 M€/y, depending on the assumption on the different pollutant-outcome relationships [27]. DIDEM was also recently used to evaluate the future expansion of the DH network of Turin [28], alternative locations of the power plants [29], and the application of different pollutant dispersion models [30]. 

Centralized heating systems, however, do not generally cover the demand of domestic hot water (DHW), which is produced individually in each property with dedicated gas or electric boilers. Electric boilers are critical for several factors, among which are the high emissions of GHG for electricity production and the intensive exploitation of the available power, i.e., about 1 kW on the typical 3 kW residential utility. On the other hand, domestic hot water (DHW) gas boilers produce local-scale pollution due to their NOx and CO emissions. The share of DHW on the total thermal energy demand of building is expected to increase in the future, since the space heating demand has large margins of reduction with better insulation of walls and windows [12], while the demand of DHW has lower margins for reduction (personal habits, dish washers, energy saving water taps, and shower heads) [31]. 

Heat pumps for buildings are growing fast in Europe, from 2.2 million units in 2007 to 9.5 million in 2016 (+17.6% yearly growth) installed in European Union [32]. Most of the heat pumps installed are reversible heating and cooling, and no statistic is available on the share of heat pumps including DHW production. However, DHW-dedicated heat pumps have recently grown fast: the 11.2% yearly increase pace from 2007 to 2011 (from 179,000 units installed to 274,000 in 2011) has recently doubled (22.1% yearly from 274,000 in 2011 to 743,000 in 2016) [32].

In this work, the benefits of replacing DHW gas and electric boilers with heat pumps are comprehensively assessed from the point of view of avoided emissions of air pollutants at the local scale and on the monetary impact that this would have on the health of the population. Although it is not the focus of this work, the financial aspect of replacing existing DHW systems with heat pumps is addressed as well, in order to assess the economic feasibility of the proposed solution. 

Alternatives to heat pumps to replace gas boilers are solar thermal panels and electric water heaters, both of them with no pollutant emissions on site. However, the technical feasibility of installing solar thermal panels is hampered by several factors, such as space availability to install enough panels to cover the whole demand, the need to install a centralized DHW system exploiting the common roof space, and restrictions on historical buildings. Water electric heaters were not considered in this study as they significantly increase overall emissions and costs for DHW production.

The paper is structured as follows. Section 2 presents the input data of estimated DHW demand in the municipality of Turin, identifying the share which is covered by dedicated boilers (and not by a combined space heating-DHW boiler), in order to derive the NOx and CO emissions which would be avoided by introducing heat pumps (Section 2.1). The air dispersion model implemented in SPRAY is presented in Section 2.2, whereas a synthesis on the DIDEM model is presented in Section 2.3. The method used for the financial assessment is presented in Section 2.4. Section 3 presents the results in terms of spatial distribution of NOx and CO concentration (Section 3.1), comparing them with data from the air quality monitoring network (Section 3.2), and assessing health externalities with the DIDEM model (Section 3.3). The financial feasibility assessment is presented in Section 3.4. Section 4 reports the conclusion of this work. 

## 2. Input Data and Methodology

Environmental impacts of NOx and CO produced by DHW gas boilers were studied in the municipality of Turin for the year 2015. At first, data concerning the type of DHW heat plants (centralized or autonomous) installed in every dwelling and the employed energy source were retrieved. This information, together with population spatial distribution, was used to calculate the DHW demand of Turin’s population and subsequently, the NOx and CO mass discharge emitted by gas boilers (Section 2.1). These discharge values were divided into 184 virtual areal square sources (one per km^2^ of surface) based on the spatial distribution of population in Turin, thus deriving the input for the air dispersion model (Section 2.2). The resulting air pollutant concentrations represent the input of a public health impact model (Section 2.3). Eventually, a financial cost-benefit analysis is carried out in Section 2.4 to assess the economic feasibility of replacing gas boilers with DHW air-source heat pumps.

### 2.1. Assessment of DHW Demand for Dedicated Gas Boilers

In view of reducing the impact of DHW production separately from space heating, the different options for DHW production should be considered, namely (1) production of DHW separated from space heating, in a centralized heating system (i.e., serving several building units), (2) production of DHW separated from space heating, in an autonomous heating system (i.e., serving one building unit), (3) production of DHW combined with space heating, in a centralized heating system, and (4) production of DHW combined with space heating, in an autonomous heating system.

The options 1 and 4 are the most frequent and our analysis focused on option 1, since this is the case for which the replacement of the DHW production unit is easier and cheaper, with no intervention required on the heating system. As described below, the most common choices for dedicated DHW production appliances are gas boilers and, to a lesser extent, electric water heaters, whereas heat pumps are still rarely used. Our analysis focused on building units where DHW is produced with a dedicated gas boiler because the aim of this work is to assess the avoidable air quality impact of DHW production.

**Figure 1:**
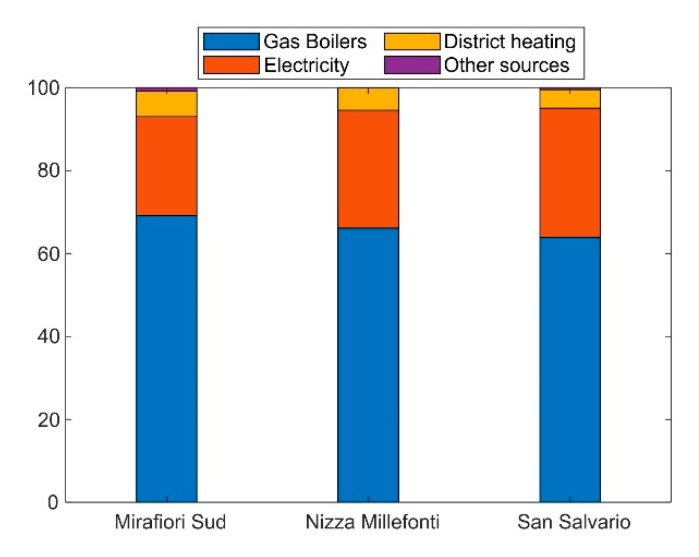
Breakdown of domestic hot water (DHW) production techniques: gas boilers (dedicated to DHW or combined with heating), electrical water heater, district heating, and other techniques.

Statistics on DHW production techniques in Turin were retrieved from the regional information system on building energy assessment [33], for the districts Mirafiori Sud (34,960 inhabitants), Nizza Millefonti (34,330 inhabitants), and San Salvario (38,110 inhabitants). Together, these districts represent 12% of the population of the municipality of Turin. The SIPEE database contains information on any building unit sold, rented, or refurbished since 2009. Statistics were therefore derived on autonomous and centralized heating plants, on heating and DHW production techniques, and energy sources (gas boilers, liquefied petroleum gas (LPG) boilers, oil boilers, district heating, electrical water heaters, electrical water heater, heat pumps). As shown in Figure 1, the shares of each energy source used for DHW production are consistent among different districts, with largely prevailing gas boilers (>60%), a noticeable use of electrical water heaters (despite their high operational cost), and a minor share of district heating and other sources. Electrical water heaters are adopted for separate DHW production, but they have not been considered in our study since their use is not deemed to affect local air quality like gas combustion does. 66% of building units in Mirafiori Sud have a separate gas boiler for DHW production and this share was considered as representative of all of Turin. A confirmation of the adequacy of this assumption is given by statistics on the district heating network of Turin, which has reached 68.9 Mm^3^ of served heated volume, i.e., 57% of the total heated volume of the city [26].

The emissions were calculated starting from an assumed DHW need ($V_{DHW}$) of 50 L/d at 45 °C, starting from a temperature of 15 °C. The individual energy need for DHW is therefore:

(1) $$Q_{DHW}={\left(\rho c\right)}_{w}\cdot V_{DHW}\cdot \Delta T$$

where ${\left(\rho c\right)}_{w}$ is the heat capacity of water equal to 1.162 kWh/(m^3^K), and ΔT is the temperature difference to be applied, i.e., 45 − 15 = 30 °C. 

Based on the assumptions above, the DHW energy need is equal to 636 thermal kWh (hereafter, kWh_th_) per year per capita and the overall DHW demand to be covered by heat pumps is 377.6 GWh_th_/year for the population considered for the calculation (593,533 people, i.e., 66% of the population of Turin). The GHG emission factor for gas boilers reported in Ref. [10] is of 235.6 g CO_2_ equivalent per thermal kWh (hereby, gCO_2_ eq./kWh_th_). The GHG emission factor of DHW heat pumps is equal to 101.3 g CO_2_ eq./kWh_th_ as it is obtained by assuming the GHG emission factor of the Italian electrical grid (303.9 gCO_2_ eq. per electrical kWh, hereby kWh_el_; see Ref. [34]) and a seasonal performance factor SPF = 3. DHW heat pumps reduce GHG emissions of 57%, which corresponds to 85 kg CO_2_ eq./person/year and to a total of 50,711 t CO_2_ eq./y avoided.

Regarding air pollution, emission factors of 80 mg NOx/kWh_th_ and 90 mg CO/kWh_th_ were assumed for the boilers to be phased out. This is a cautious assumption, since such values are typical of up-to-date boilers. As explained in the introduction, gas boilers have a slow renewal rate and hence the emission factors of boilers to be replaced by heat pumps is likely to be much higher [6]. The resulting overall local-scale mass flow rate of air pollutants is of 30.2 tNOx/y and 34 tCO/y, and it was implemented in the air dispersion model (Section 2.2) as 184 virtual areal sources, one per each km^2^, based on the population density in the Turin municipality, which is known with the same resolution.

Although the objective of this work is a local-scale analysis of pollutant emissions, some consideration should also be given to overall pollutant emissions (due to electricity production) when replacing DHW gas boilers with heat pumps. Casasso et al. [10] recently provided pollutant emission factors for the Italian electrical grid. As of 2016, the global NOx emission factor of a DHW HP is similar (−1% compared to a gas boiler), whereas a strong reduction is achieved for CO (−64%). However, it should be observed that NOx from the Italian electrical grid is decreasing at a fast rate (−18% between 2010 and 2016 according to SINAnet [35]) as the share of cleaner energy sources (solar photovoltaics, wind, hydropower, up-to-date gas power plants) are replacing dirtier ones such as coal (which is going to be phased out in 2025, see Reference [36]). In addition, NOx concentrations are critical in urban areas and, hence, displacing NOx out of them results in a significant reduction of health externalities.

A strong concern for air-source heat pumps is represented by noise [37,38,39]. Although this holds true for large-power HPs (e.g., 50 kW and above, which can exceed sound power level of 60 dB at 10 m), DHW HPs have a much lower thermal power (1–2 kW) and hence a much lower sound power level (50 dB at the source, e.g. see Reference [40]). This noise can easily be abated by a wall, both for an indoor (e.g., bathroom) and an outdoor (e.g., balcony) installation.

### 2.2. Air Dispersion Modeling

The dispersion of NOx and CO from DHW gas boilers was performed using the numerical code SPRAY [41], a commercial three-dimensional Lagrangian particle dispersion model licensed by Arianet company [42]. SPRAY takes into account the spatial and temporal inhomogeneities of both average flow and turbulence to calculate concentration fields generated by point, area, or volume sources. The trajectory of the airborne pollutant is simulated through virtual particles: the mean motion is defined by the local wind and the dispersion is determined solving the Langevin stochastic differential equations for the velocity fluctuations, reproducing the statistical characteristics of the turbulent flow. Therefore, different portions of the emitted plumes can undergo different atmospheric conditions. SPRAY thus performs a realistic representation of complex phenomena such as low wind-speed conditions, strong temperature inversions, flow over topography, land use, and terrain variability. 

In this study, gas boilers for DHW production were simulated as area sources, one per square kilometer. A total of 184 sources was obtained, following the borders of the municipality, which has a surface of 130 km^2^. The modeling domain was divided into cells of 1000 × 1000 m as well, with a total size of 101 × 101 cells. The overall annual emission of each cell was modulated based on monthly, weekly, and daily profiles provided by the norm UNI EN 16147:2017 issued by the Italian Organization for Standardization (UNI) and the European Committee for Standardization (EN) [43]. An average height of 25 m was assigned to sources, with a random variation between 20 and 30 m. 

NOx and CO emissions were modeled for a 1-year period with a time resolution of 1 hour, using the latest meteorological input datasets (year 2015) delivered by the Regional Agency for Environmental Protection of the Piedmont Region (ARPA) [44]. Such a dataset includes land use categories, altimetry, wind speed and direction (at ground and vertical observations), temperature (at ground and vertical observations), air pressure, air humidity, precipitation, and solar radiation. Weather and orographic data have a spatial resolution of 1000 m and cover a domain of 100 × 100 km. Such a domain extension is adequate to consider the main orographic features of the area, i.e., the hills located next to the eastern side of Turin urban area and the Alps, located approximately 30 km westwards. Meteorological data were pre-processed with the Stationary Wind Field and Turbulence (SWIFT) diagnostic mass-consistent model, developed by Aria Technologies [45].

The output obtained from dispersion modeling are 8760 grids (one per hour) of NOx and CO concentrations. Such grids were introduced in a post-processing module generating spatial distributions of the annual average concentrations and the maximum hourly and daily-averaged concentrations of NOx and CO. These data were finally transferred to the DIDEM module calculating health effects and costs.

### 2.3. Health Externalities: the DIDEM Model

The evaluation of health externalities due to air pollution can be performed with several models and tools. A detailed review was recently performed by Anenberg et al. [46], which analyzed and compared the input and output detail level of 12 air pollution health impact assessment tools. The DIATI Dispersion and Externalities Model (DIDEM) [27], which builds on these tools, was conceived to quantify and minimize the overall uncertainty thanks to the integration of (i) advanced pollutant dispersion models with the calculation of health concentration-response functions (CRFs), implemented following the latest World Health Organization (WHO) recommendations, and (ii) different confidence levels on CRFs data reported by the WHO, resulting in a precise estimation of uncertainty associated to the calculation of health effects. 

In this work, DIDEM was used to perform analyses of external health impacts and costs at the local scale (the territory of the municipality of Turin). The main output provided by the model is the variation (reduction or increase) of external costs associated to the alternative scenarios. The term “external costs” refers here to the marginal health damage costs, i.e., those costs generated by the effects on human health resulting from an extra unit of pollutant concentration.

The DIDEM model is based on the impact pathway approach [47], which links the modeled pollutant concentrations to the concentration-exposure-response functions (CRFs) provided by latest WHO recommendations of 2013 [48,49]. Monetary values are associated to the incremental incidence of disease calculated. A preliminary uncertainty estimation is achieved through the implementation of different confidence levels on CRFs data and the application of confidence intervals in the calculation of the slope of the concentration-response function. Confidence levels are based on the recommendations reported in the WHO Health Risks of Air Pollution In Europe (HRAPIE) project [48], where the pollutant-outcome pairs are classified into Group A (pollutant-outcome pairs for which enough data are available to enable reliable quantification of effects) and Group B (pollutant-outcome pairs for which there is more uncertainty about the precision of the data used for quantification of effects). The same report provides slope values of the CRFs for NOx and CO. The effect of these contaminants is deemed linear with the air concentration and hence, the absorbed dose.

The delta-external costs are calculated as:

(2) $$C_{i,r}=\underset{r}{\sum }\underset{i}{\sum }\left[\Delta c_{r}\times p_{r}\right]\times s_{CRi}\times m_{i}$$

where $C_{i,r}$ represents the damage costs related to pollutant outcome pair *i* and to domain cell *r*, (€), $\Delta c_{r}$ is the concentration change (μg/m^3^) of a given pollutant, referred to domain cell *r*, and $p_{r}$ is the number of exposed individuals, $s_{CRi}$ is the slope (additional cases/((μg/m^3^) · person · year)) of the impact function of health impact *i*, merging information on the risk increase and baseline rate of a given health impact *i*, and $m_{i}$ is the monetary value (€/additional case) per case of health impact *i*. For the complete list of pollutant-outcome pairs considered, refer to Reference [15]. 

Monetary values per case of health impact (*m_i_* in Equation (2)) of EU countries (or Regions) are implemented in the model. These data were taken by the most recent updates issued for the EU Clean Air Package [49]. Monetary values are converted to the reference year using an average EU inflation rate of 2.1% [50].

### 2.4. Financial Evaluation

Although the main aim of this work was to evaluate the air quality benefits of replacing DHW gas boilers, a financial evaluation was performed to prove the feasibility for the Turin citizens who are expected to replace their boilers. The evaluation was performed by calculating the internal rate of return (IRR) over the lifetime of a heat pump (assumed as 25 years), with the further assumption that no replacement of the gas boiler was necessary in the same period. The heat pump purchase was therefore considered as an investment that is repaid by savings in DHW production and the incentives for energetic refurbishment of buildings.

The IRR is defined as the discount factor r that, during the period considered (in this case, 25 years), makes the discounted net present value (DNPV) null:

(3) $$DNVP_{25}=-INV+\overset{25}{\underset{i=1}{\sum }}\frac{\Delta M\left(i\right)+\Delta O\left(i\right)+Inc\left(i\right)}{{\left(1+r\right)}^{i}}$$

where INV is the initial investment (i.e., the installation cost of the heat pump), ΔM is the difference in maintenance costs between the replaced device and the new one, ΔO is the difference of the operative costs (i.e., the production of DHW), and Inc is the incentive. All these variables are monetary (€). 

For the sake of simplicity, the difference in maintenance costs (ΔM) was assumed null, although various literature sources state that heat pumps have lower maintenance costs compared to gas boilers (e.g., no flue gas control is required, less clogging issues, etc.). Also, the difference of operation costs ΔO for DHW production was considered as constant during the period analyzed. 

As for the incentives, the Italian regime for residential energetic refurbishment was considered, i.e., the refund of 65% of the installation costs incurred, with 10 yearly payments of 6.5% each [51]. 

With the premise above, Equation (3) changes to:

(4) $$DNVP_{25}=-INV+\overset{10}{\underset{i=1}{\sum }}\frac{\Delta O+0.65\cdot INV}{{\left(1+r\right)}^{i}}+\overset{25}{\underset{i=11}{\sum }}\frac{\Delta O}{{\left(1+r\right)}^{i}}$$

The IRR is the solution of the following equation:

(5) $$\overset{10}{\underset{i=1}{\sum }}\frac{\Delta O+0.65\cdot INV}{{\left(1+IRR\right)}^{i}}+\overset{25}{\underset{i=11}{\sum }}\frac{\Delta O}{{\left(1+IRR\right)}^{i}}=INV$$

The initial investment was quantified based on a market analysis on heat pump costs from different manufacturers [52,53,54], hypothesizing different needs of consecutive showers (with 50 liters per each shower) depending on the number of cohabitants and considering different tank volumes, as showed in Table 1. Generally, wall-mounted heat pumps are available up to 200 L tanks, while larger ones need to be installed on the floor. Both mono-block and split heat pumps are available, each kind suitable for different setups, yet their prices are similar, and a proper choice can be made depending on the characteristics of the building unit. The most influential variable was found to be the tank size, with a linear variation of about 8 €/L tank up to 300 L, and a smaller increment (about 4 €/L tank) between 300 L and 500 L. A flat installation cost of 500 € was assumed for all heat pump sizes.

DHW production costs were derived using input data by the European Statistical system (EUROSTAT) [55], i.e., 0.095 €/kWh_th_ for gas and 0.21 €/kWh¬_el_ for electricity. The boiler efficiency was assumed equal to 90% and the heat pump SPF was assumed equal to 3. The DHW cost is therefore equal to 0.1055 €/kWh_th_ for the gas boiler and of 0.07 €/kWh_th_ for the DHW heat pump, with a cost reduction of 33.6% achieved by the heat pump compared to the gas boiler. The savings margin with the heat pump is therefore 22.60 €/person/year.

Table 2 summarizes the characteristics of gas boilers and of DHW HPs considered in this work.

## 3. Results

With the methods described in the previous Section, the costs and benefits of replacing gas boilers (dedicated to DWH production only) with heat pumps were assessed from different points of view, which are hereby analyzed. Section 3.1 presents the results of the air dispersion modeling of the currently installed DHW gas boilers. Section 3.2 presents the comparison of modeled concentrations time series at two locations, where air quality monitoring stations are present. Section 3.3 presents the results on the modeling externalities on human health. Finally, Section 3.4 presents the financial feasibility assessment of replacing gas boilers with heat pumps.

### 3.1. Pollutant Emissions Due to DHW Production 

**Figure 2:**
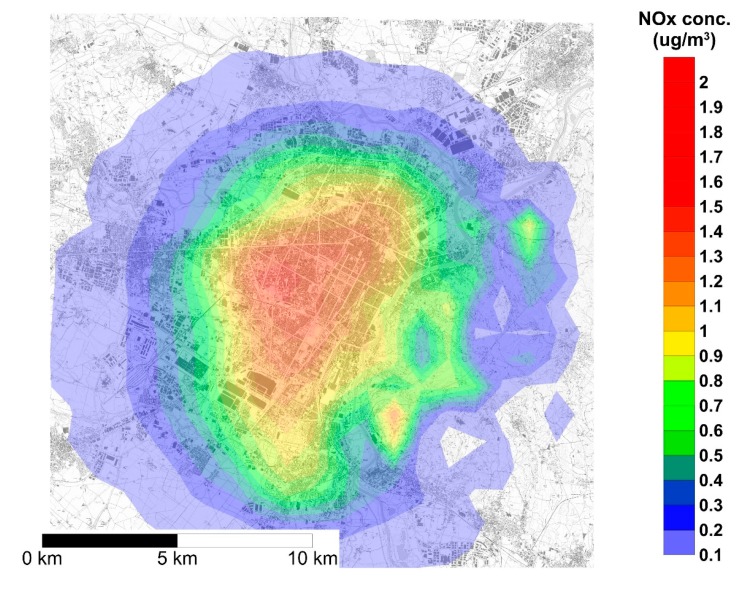
Yearly 1 h average concentration of NOx over the urban area of Turin generated by the emission of natural gas boilers for domestic hot water (DHW) production.

**Figure 3:**
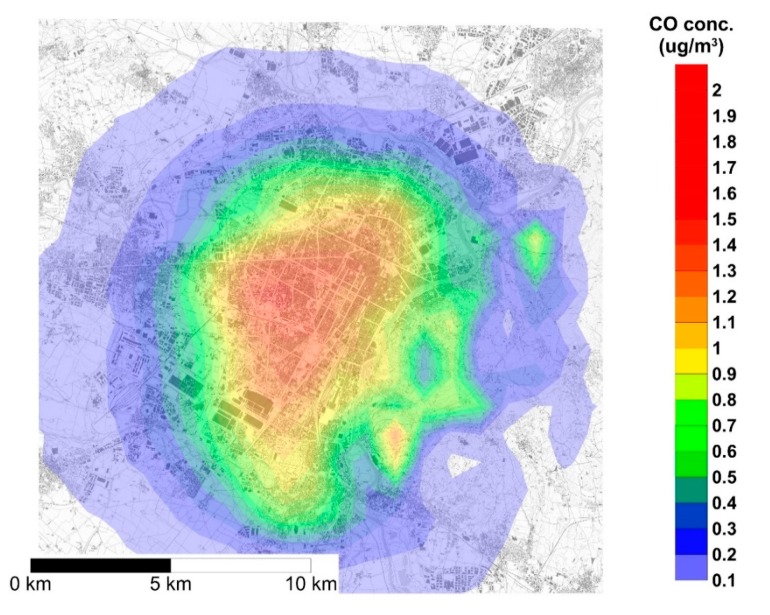
Yearly 1 h average concentration of CO over the urban area of Turin generated by the emission of natural gas boilers for domestic hot water (DHW) production.

The map reported in Figure 2 shows the spatial distribution of the yearly average NOx concentrations generated by the DHW gas boilers to be replaced by heat pumps and, hence, the average concentration reduction consequent to such replacement. The NOx yearly average concentration in the urban area of Turin ranges from 0.1 μg/m^3^ to 1.4 μg/m^3^. Since the model implemented in SPRAY does not reproduce reactions, the spatial distribution of yearly average CO concentration has a similar pattern, as shown in Figure 3, with concentrations ranging from 0.1 μg/m^3^ to 1.7 μg/m^3^. Figure 2 and Figure 3 show that pollutant dispersion is mainly limited to the urban area and to the hilly areas located in the eastern part of the town. Such a limited spatial dispersion is mainly due to the combined effect of the low height of the sources, and meteorological features of the area such as low winds. 

**Figure 4:**
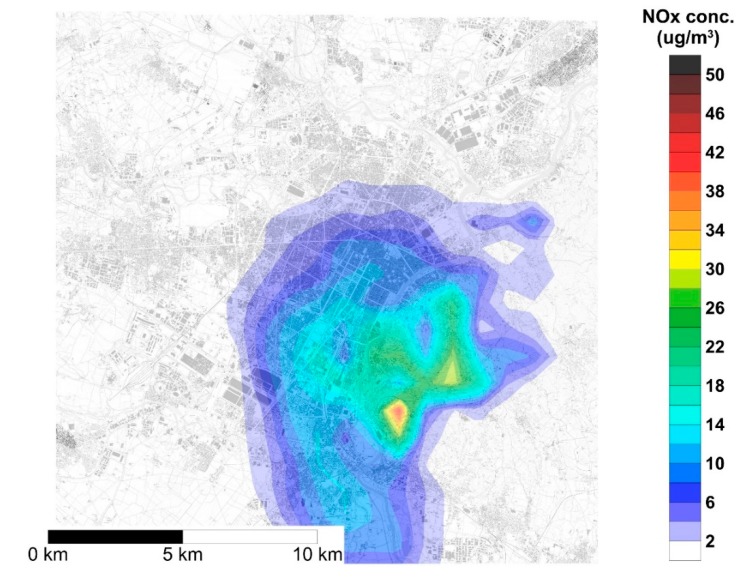
Map of NOx concentration over the urban area of Turin corresponding to the occurrence of the maximum average concentration on the modeling domain (Day: 8 December, 0:00 a.m.).

**Figure 5:**
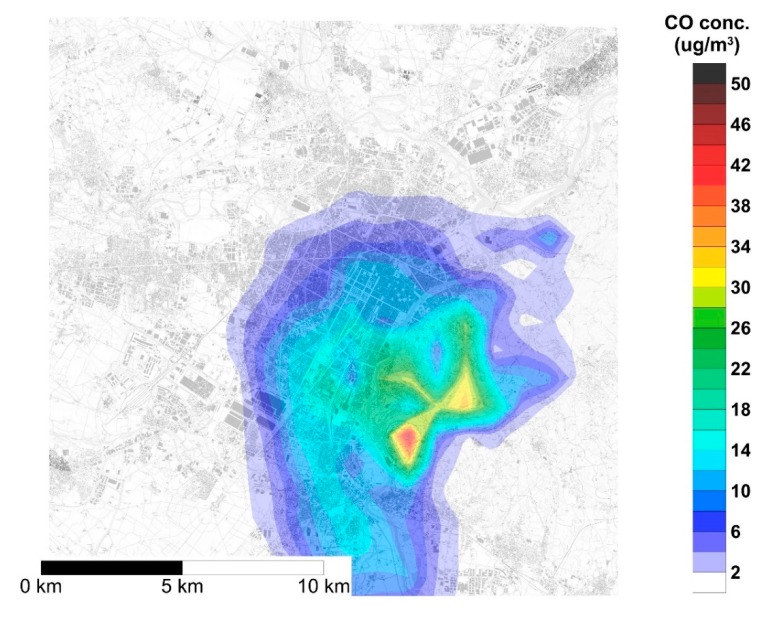
Map of CO concentration over the urban area of Turin corresponding to the occurrence of the maximum average concentration on the modeling domain (Day: 8 December, 0:00 a.m.).

In Figure 4 and Figure 5, the concentration maps corresponding to the maximum hourly concentration are reported for NOx and CO, respectively. These maps were generated by calculating the average concentration over the entire modeling domain, then extracting the maximum of hourly observations, which occurred at 0:00 of 8 December. These figures show that, under critical meteorological conditions, NOx concentration due to DHW production by gas boilers can reach up to 30–40 μg/m^3^, with the highest concentrations on the hills on the east of the city center. 

**Figure 6:**
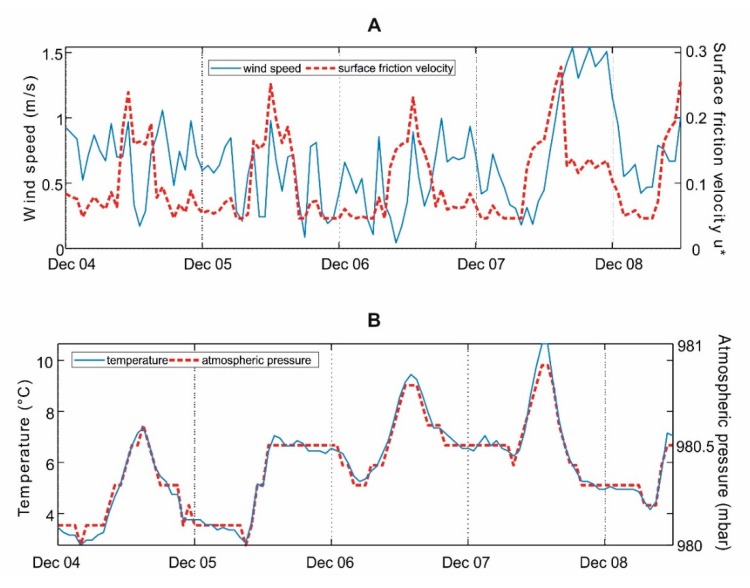

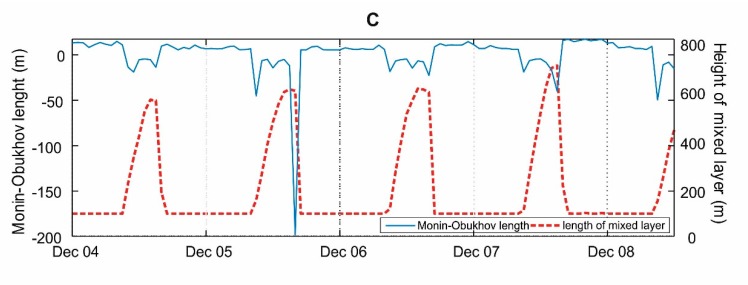
Meteorological conditions in correspondence of the maximum average hourly concentration on the area, occurred at 0:00 of day 8 December: (**A**) Wind speed and surface friction velocity, (**B**) temperature and atmospheric pressure, and (**C**) Monin–Obukhov length and height of the mixed layer.

The meteorological parameters measured in the area during the four days preceding the event of maximum concentration are reported in Figure 6. The maxima of NOx and CO concentrations were observed in conditions of very low wind speed (<1 m/s), limited surface friction velocity, low temperature and atmospheric pressure, Monin–Obukhov length values typical of prevailing stable atmospheric conditions, and limited height of the mixed layer (below 700 m during the whole period). Such conditions clearly explain the accumulation of pollutants in the hills area on the east. 

### 3.2. Assessment of the Relative Influence of DHW Production on NOx and CO Emissions and Concentrations

The concentrations resulting from air dispersion modeling were compared to those observed at the air quality monitoring stations, in order to assess the relative influence of DHW production on air pollution in Turin. Air quality monitoring is managed by the regional environmental protection agency (ARPA Piemonte, Italia). The monitoring stations of Consolata (city center) and Rebaudengo (northern outskirts) were chosen, because they are located in the most densely populated area where the highest concentrations were resulting from the model. Records for the year 2015 were chosen for the comparison, since this is the same year used for the meteorological model.

**Figure 7:**
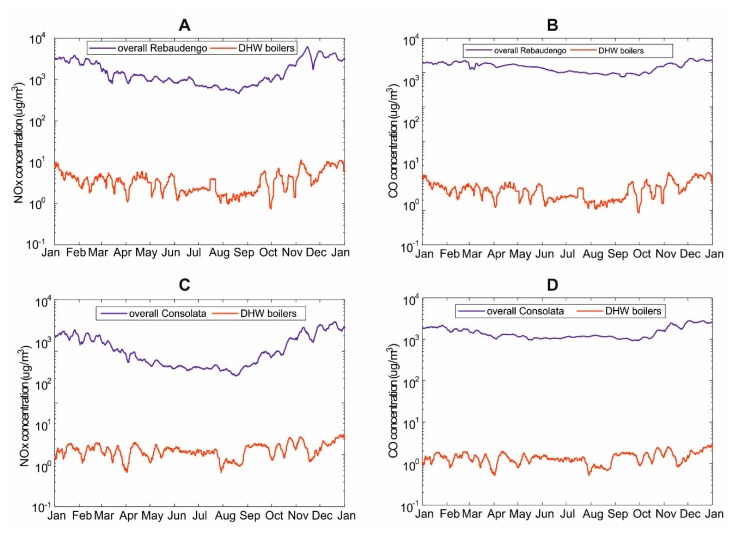
Comparison of concentrations of NOx (**A** and **C**, on the left) and CO (**B** and **D**, on the right) measured at the monitoring stations of Rebaudengo (**A** and **B**, on the top) and Consolata (**C** and **D**, at the bottom) with the corresponding time series of the modelled concentrations.

Figure 7 reports such a comparison using the seven-day averaged concentration values of NOx (Figure 7A–C) and CO (Figure 7B–D) modeled with SPRAY at the location point of Rebaudengo, in comparison with the corresponding records of the ARPA station. The values of yearly average NOx concentrations exhibit a difference of nearly two orders of magnitude between the overall value (176.58 μg/m^3^ and 124.97 μg/m^3^ respectively, at Rebaudengo and Consolata) and the share, modeled with SPRAY due to the DHW gas boilers (0.38 μg/m^3^ and 1.25 μg/m^3^, i.e., 0.2% and 1.1%, respectively) (Figure 7A–C). Such a difference is even larger for CO concentrations, with approximately three orders of magnitude between the yearly average concentrations at the air monitoring stations (1480.57 μg/m^3^ and 1478.36 μg/m^3^ respectively, at Rebaudengo and Consolata) compared to the modeled concentrations (0.43 μg/m^3^ and 1.41 μg/m^3^, i.e., 0.03% and 0.09% of total CO concentrations) (Figure 7B–D).

Based on these evidences, DHW production by gas boilers has an appraisable—but not dramatic—impact on the air quality of Turin. 

The comparison performed is rather consistent with the estimates of the air emission inventory INEMAR [56], which estimated 5204 tNOx/y and 10,404 tCO/y emitted in the municipality of Turin in 2013: the share of emissions assumed for DHW gas boilers (see Section 2.1) are, respectively, 0.58% and 0.32%.

### 3.3. Health Externalities

**Figure 8:**
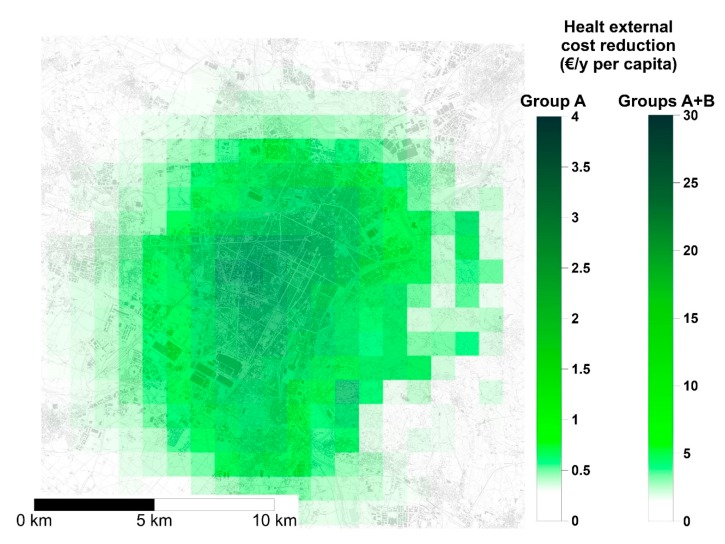
Distribution of external costs (€/y per capita) over the urban area of Turin following the replacement of natural gas boilers for domestic hot water (DHW) production. Pollutant-outcome pairs of Group A (high confidence) and Group A + B (high and medium confidence).

The spatial distribution of health externalities due to gas DHW boilers, evaluated with DIDEM as explained in Section 2.3, is reported in Figure 8. Data are reported in €/y per capita to allow a comparison of health externalities among differently populated areas of the city. The area with the highest external cost reduction corresponds to the urban center of Turin. 

Considering the pollutant-outcome pairs of Group A, the installation of heat pumps for DHW production would bring a cost reduction of 0.4 ÷ 2.8 €/y per capita; however, if Group B is also included, such unit cost increases up to 20 €/y per capita (Figure 8). 

Results calculated on the whole population of Turin are reported in Table 3. The estimation of total external cost reduction is differentiated depending on the implementation of different confidence levels on CRFs data (Group A and Group A + B) and the application of confidence intervals in the calculation of the slope of the concentration-response function (minimum, mean, maximum). If pollutant-outcome pairs with high confidence level on CRF data are considered (Group A), total external costs reduction ranges from 1.05 M€/y to 2.6 M€/y, with a mean value of 1.85 M€/y. If pollutant-outcome pairs with both high and medium confidence level on CRF data are considered (Group A + B), total external costs reduction ranges from 5.75 M€/y to 15.15 M€/y, with a mean value of 10.4 M€/y.

### 3.4. Financial Analysis 

The financial feasibility of replacing DHW gas boilers with heat pumps was performed at two different scales: the single residential unit and the whole population considered for boiler replacement (593,533 people). 

**Figure 9:**
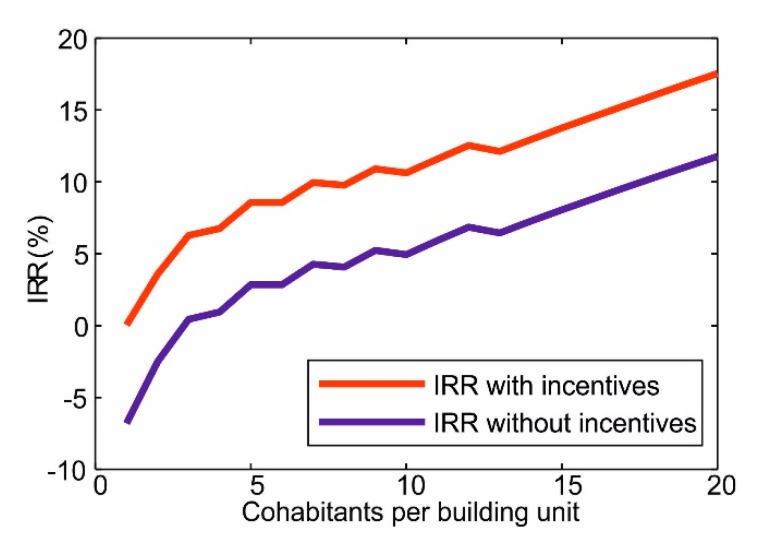
Internal Rate of Return (IRR) value with and without considering the contribution of incentives.

At the scale of a single residential unit, the financial feasibility depends on the number of cohabitants. As reported in Section 2.4, based on unit costs of DHW production, a saving margin of 22.60 €/y per capita is calculated. The initial investment required to install a DHW heat pump depends on the number of cohabitants of each building unit, as reported in Table 4. The internal rate of return (IRR) of installing a DHW HP was calculated for building units from 1 to 20 people, over a heat pump lifetime of 25 years, using Equation (5). Results are reported in Figure 9, showing an increasing trend of the IRR (both with and without incentives) as the number of cohabitants increase. This confirms the presence of scale economies in replacing DHW gas boilers. In the presence of incentives, DHW HP are always convenient with IRR ranging between 0.04% and 17.54%, with a steep increase from 1 to 3 cohabitants due to the economies of scale. In the absence of incentives, negative IRR values are found for building units with 1 or 2 cohabitants.

The extension of the financial analysis to the whole city of Turin should consider the sharing of building units with different numbers of cohabitants. According to the Yearbook 2011 of the Turin Municipality [57], 21% of people live in single residential units, 27.8% live in flats with 2 cohabitants, 23.9% with 3 cohabitants, 19.7% with 4 cohabitants, and 7.6% in flats with 5 or more cohabitants.

Assuming the residential units involved in the DHW boiler replacement have the same breakdown, the financial return of installing DWH HPs was performed. The total investment needed to perform such replacement is of 483.11 M€. However, most of this sum would be needed to replace DWH boilers in residential units with 1 or 2 cohabitants (respectively, 41.2% and 27.3% of the total investment needed). The minimum (1.05 M€/y) and the maximum (15.15 M€/y) values of health externalities, calculated in Section 3.3, were used to perform the overall financial analysis reported in Table 4. 

Including the health externalities in a city-scale financial evaluation, the replacement of DHW gas boilers is always convenient, except for the case when the lowest value of health externalities (1.05 M€/y) is considered. The scenarios hypothesizing the absence of incentives for energetic refurbishment, which would cover 65% of the investment made by Turin citizens (i.e., 314.02 M€ on a total of 483.11 M€), were considered to assess the effect of a phase-out of such incentives: depending on the hypotheses adopted to calculate the health externalities, the overall balance could still be positive; however, DHW gas boiler are not likely to be replaced by citizens if such a choice is not economically convenient for them. 

A final evaluation was made on how the population of Turin would benefit from a partial replacement of DHW gas boilers. As shown in Figure 9, the convenience of installing a DHW HP increases as the number of cohabitants of the residential unit increases, with a sharp increase from 1 to 3 cohabitants. This means that, from a merely financial point of view, priority should be given to replacing DHW boilers in more populated building units, while single ones are the least convenient. In addition, key quantities related to the advantages of introducing DHW HPs—the savings on DHW production, the avoided greenhouse gas emissions, the avoided air pollutant emissions—are proportional to the number of people served (and, hence, the DHW produced) by DHW HPs instead of gas boilers. In addition, if one considers a distribution of people served by DHW HP which perfectly agrees with the distribution of population density, the reduction of NOx and CO concentration and health externalities are also proportional to the overall number of people served by DHW HPs.

**Figure 10:**
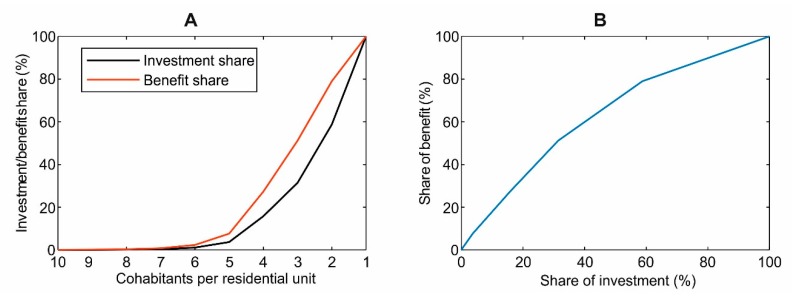
Correlation between the share of the total investment (486.88 M€) and the share of total benefits (emissions avoided: 30.2 t NOx/year, 34 t CO/year, 55,701 tCO2/year; reduced domestic hot water (DHW) production costs: 13.41 M€/y; avoided health externalities: 1.05 ÷ 15.15 M€/y): (**A**) correlation of investment and benefits achieved, with the decreasing number of cohabitants per housing unit in which DHW gas boilers are replaced, and (**B**) correlation between the share of the total investment and the share of benefits achieved.

Therefore, considering the replacement of gas boilers separately, all units with decreasing number of cohabitants (i.e., from 20 to 1) provides an estimate on how the overall benefits increase with the spent share of the overall investment (i.e., 483.11 M€). As shown in Figure 10, the marginal gain of replacing gas boilers diminishes as this is less and less populated housing units. For example, replacing DHW gas boilers in all units with 4 or more cohabitants results in 27.33% of the overall economic and health benefit, with 15.8% (76.29 M€) of the total expense. On the other hand, introducing DHW HPs in single housing units would require 41.2% (199.16 M€) of the overall investment and would lead to only 21% of the overall benefit in terms of DHW cost reduction, GHG and air pollutant emissions, and health externalities avoided.

## 4. Conclusions

Although they still represent a minor market niche, domestic hot water heat pumps (DHW HPs) have been increasing fast in recent years in Europe, following the general trend of heat pumps and other renewable heating techniques. DHW HPs reduce the economic cost and the greenhouse gas emissions due to domestic hot water. In addition, a massive diffusion of DHW HPs in urban areas can provide an appraisable contribution to the improvement of air quality.

We presented a study focused on the city of Turin (NW Italy) to assess the reduction of NOx and CO emissions potentially achievable by replacing all gas boilers dedicated to DHW production. Such boilers were first identified based on a statistic study, estimating that about 66% of Turin’s population lives in residential units with a centralized heating system, where a gas boiler is used for DHW production. Gas boilers used also for space heating were not considered in our study, since their replacement would require more expensive heat pumps and massive intervention on heating terminals.

The DHW which need to be covered by heat pumps is estimated at 377.6 GWh/y. With a safe-side assumption of replacing boilers with a low emission factor for NOx (80 mgNOx/kWh) and CO (90 mgCO/kWh), a total avoided emission of 30.2 tNOx/y and 34 tCO/y was estimated. In addition, a reduction of 55701 t CO_2_ eq_._/y was estimated for GHG.

The air dispersion of such pollutants was modelled with the code SPRAY, considering a space distribution of pollutant sources with a mass flow rate proportional to the population density. The yearly average modeled concentrations of pollutants achieve maximum values of 1.4 µg NOx/m^3^ and 1.7 µg CO/m^3^, respectively. Comparing yearly average concentrations at two ARPA monitoring stations (Consolata and Rebaudengo), the impact on NOx concentrations is of 0.2% ÷ 1.1%, whereas the CO is far less impacted (0.03% ÷ 0.09%).

The financial feasibility of replacing DHW gas boilers with heat pumps was also evaluated. It turns out that DHW HPs are always financially feasible exploiting the current Italian incentives (65% on the total investment), whereas, in the absence of incentives, DHW HPs are convenient only for dwellings with 3 people or more (i.e., 51.22% of the population) thanks to economies of scale. This study provided a comprehensive evaluation of the environmental benefits of replacing DHW gas boilers with heat pumps, providing a methodology which could be replicated for other urban areas. 

## Figures and Tables

**Table 1:** Heat pump tank sizes and prices, depending on the number of cohabitants of a building unit.

Cohabitants	Tank Size (L)	HP Cost (€)	Investment (€)
1–3	100	1100	1600
4–5	150	1500	2000
6–7	200	1900	2400
8–9	250	2300	2800
10–12	300	2700	3200
13–20	500	3100	3600

**Table 2:** Summary comparison of the characteristics of a gas boiler and a heat pump for domestic hot water (DHW) production.

Domestic hot water (DHW) Device	Fuel Consumption (Per Year, Per Capita)	Thermal Power of a Unit (kW_th_) (Typical Values)	Energy Efficiency	Energy Cost (€/kWh_th_)
Gas boiler	73.69 m^3^ gas	30	90%	0.1055
Heat pump	212 kWh_el_	0.8 ÷ 2	COP = 3	0.0700

**Table 3:** Total reduction of health externalities over the studied area following the replacement of natural gas boilers for domestic hot water (DHW) production.

Confidence Level on CRF data	Delta External Costs mean (M€/y)	Delta External Costs minimum (M€/y)	Delta External Costs maximum (M€/y)
High (Group A)	−1.85	−1.05	−2.60
Medium (Group A + B)	−10.40	−5.75	−15.15

**Table 4:** Net positive values (NPV) and internal rate of return (IRR) calculated for the replacement of DHW boilers with heat pumps for the whole city of Turin, including the positive health externalities of such initiative.

Scenario	NPV (M€)	IRR
Minimum health externalities, with incentives	192.51	3.93%
Maximum health externalities, with incentives	545.01	8.97%
Minimum health externalities, without incentives	−121.52	−2.11%
Maximum health externalities, without incentives	230.98	3.26%

## References

[1] ICCT A Technical Summary of Euro 6/VI Vehicle Emission Standards | International Council on Clean Transportation. http://bit.ly/3ag3l7l.

[2] Ürge-Vorsatz D., Cabeza L.F., Serrano S., Barreneche C., Petrichenko K. (2015). Heating and cooling energy trends and drivers in buildings. Renew. Sustain. Energy Rev..

[3] Kheirbek I., Haney J., Douglas S., Ito K., Caputo S., Matte T. (2014). The Public Health Benefits of Reducing Fine Particulate Matter through Conversion to Cleaner Heating Fuels in New York City. Environ. Sci. Technol..

[4] Özden Ö., Döğeroğlu T., Kara S. (2008). Assessment of ambient air quality in Eskişehir, Turkey. Environ. Int..

[5] EUROPEAN COMMISSION Mapping and Analyses of the Current and Future (2020–2030) Heating/Cooling Fuel Deployment (Fossil/Renewables). http://bit.ly/2PxBbwf.

[6] (2016). Danish Gas Technology Center Facts and Figures about Domestic Gas Boilers. A Compilation of Results Covering 25 Years of Testing at DGC’s Laboratory. http://bit.ly/38RQO9u.

[7] Sarigiannis D.A., Karakitsios S.P., Kermenidou M.V. (2015). Health impact and monetary cost of exposure to particulate matter emitted from biomass burning in large cities. Sci. Total Environ..

[8] Crounse J.D., DeCarlo P.F., Blake D.R., Emmons L.K., Campos T.L., Apel E.C., Clarke A.D., Weinheimer A.J., McCabe D.C., Yokelson R.J. (2009). Biomass burning and urban air pollution over the Central Mexican Plateau. Atmos. Chem. Phys..

[9] Liu T., Marlier M.E., DeFries R.S., Westervelt D.M., Xia K.R., Fiore A.M., Mickley L.J., Cusworth D.H., Milly G. (2018). Seasonal impact of regional outdoor biomass burning on air pollution in three Indian cities: Delhi, Bengaluru, and Pune. Atmos. Environ..

[10] Casasso A., Capodaglio P., Simonetto F., Sethi R. (2019). Environmental and Economic Benefits from the Phase-out of Residential Oil Heating: A Study from the Aosta Valley Region (Italy). Sustainability.

[11] Saner D., Juraske R., Kübert M., Blum P., Hellweg S., Bayer P. (2010). Is it only CO2 that matters? A life cycle perspective on shallow geothermal systems. Renew. Sustain. Energy Rev..

[12] Rivoire M., Casasso A., Piga B., Sethi R. (2018). Assessment of Energetic, Economic and Environmental Performance of Ground-Coupled Heat Pumps. Energies.

[13] Becchio C., Bottero M.C., Casasso A., Corgnati S.P., Dell’Anna F., Piga B., Sethi R. (2017). Energy, economic and environmental modelling for supporting strategic local planning. Procedia Eng..

[14] Lombardi F., Rocco M.V., Colombo E. (2019). A multi-layer energy modelling methodology to assess the impact of heat-electricity integration strategies: The case of the residential cooking sector in Italy. Energy.

[15] Hedegaard K., Mathiesen B.V., Lund H., Heiselberg P. (2012). Wind power integration using individual heat pumps–Analysis of different heat storage options. Energy.

[16] Carvalho A.D., Moura P., Vaz G.C., de Almeida A.T. (2015). Ground source heat pumps as high efficient solutions for building space conditioning and for integration in smart grids. Energy Convers. Manag..

[17] Arteconi A., Hewitt N.J., Polonara F. (2013). Domestic demand-side management (DSM): Role of heat pumps and thermal energy storage (TES) systems. Appl. Therm. Eng..

[18] Lund H., Möller B., Mathiesen B.V., Dyrelund A. (2010). The role of district heating in future renewable energy systems. Energy.

[19] Lund R., Persson U. (2016). Mapping of potential heat sources for heat pumps for district heating in Denmark. Energy.

[20] Noussan M., Jarre M., Roberto R., Russolillo D. (2018). Combined vs separate heat and power production – Primary energy comparison in high renewable share contexts. Appl. Energy.

[21] Olsson L., Wetterlund E., Söderström M. (2015). Assessing the climate impact of district heating systems with combined heat and power production and industrial excess heat. Resour. Conserv. Recycl..

[22] Ravina M., Panepinto D., Zanetti M. (2018). District heating system: Evaluation of environmental and economic aspects. Int. J. Environ. Impacts.

[23] Mawdsley I., Wisell T., Håkan S., Ortiz C. Revision of Emission Factors for Electricity Generation and District Heating (CRF/NFR 1A1a). http://bit.ly/34x9Cri.

[24] Ravina M., Panepinto D., Zanetti M.C., Genon G. (2017). Environmental analysis of a potential district heating network powered by a large-scale cogeneration plant. Environ. Sci. Pollut. Res..

[25] AIRU (2019). Il Riscaldamento Urbano. Annuario 2018 (Urban. Heating. Yearbook 2018).

[26] IREN IREN-Distribuzione Calore (District Heating). https://www.gruppoiren.it/distribuzione-calore.

[27] Ravina M., Panepinto D., Zanetti M.C. (2018). DIDEM-An integrated model for comparative health damage costs calculation of air pollution. Atmos. Environ..

[28] Ravina M., Panepinto D., Zanetti M.C. (2018). A Dispersion and Externalities Model Supporting Energy System Planning: Development and Case Study.

[29] Ravina M., Panepinto D., Zanetti M. (2019). Air Quality Planning and the Minimization of Negative Externalities. Resources.

[30] Ravina M., Panepinto D., Zanetti M. (2019). Development of the DIDEM model: Comparative evaluation of CALPUFF and SPRAY dispersion models. Int. J. Environ. Impacts Manag. Mitig. Recovery.

[31] Åberg M. (2014). Investigating the impact of heat demand reductions on Swedish district heating production using a set of typical system models. Appl. Energy.

[32] EHPA European Heat Pump Market and Statistic Report 2017 & Stats Tool. https://www.ehpa.org/market-data/2017/.

[33] Regione Piemonte Sistema Informativo per la Prestazione Energetica degli Edifici (SIPEE) (Information System on Energy Performance of Builings in Piemonte Region, Italy). http://bit.ly/38bbE3n.

[34] ISPRA (2018). Fattori di Emissione Atmosferica di CO2 e altri gas a Effetto Serra nel Settore Elettrico (CO2 Emission Factors of the Electric Sector).

[35] SINANET Fattori di Emissione per la Produzione ed il Consumo di Energia Elettrica in Italia (Emission Factors for the Production and the Consumption of Electricity in Italy). http://bit.ly/2VNne2b.

[36] MISE Strategia Energetica Nazionale (SEN) 2017 (Italian National Energy Strategy 2017). http://bit.ly/30t25cj.

[37] Omlin S., Bauer G., Brink M. (2011). Effects of noise from non-traffic-related ambient sources on sleep: Review of the literature of 1990-2010. Noise Health.

[38] Aletta F., Masullo M., Maffei L., Kang J. (2016). The effect of vision on the perception of the noise produced by a chiller in a common living environment. Noise Control. Eng. J..

[39] Persson K., Rylander R. (1988). Disturbance from low-frequency noise in the environment: A survey among the local environmental health authorities in Sweden. J. Sound Vib..

[40] Ariston Nuos EVO A + 80-110 WH-Scaldacqua a Pompa di Calore | Ariston. http://bit.ly/2Rler2d.

[41] Tinarelli G., Anfossi D., Trini Castelli S., Bider M., Ferrero E., Gryning S.-E., Batchvarova E. (2000). A New High Performance Version of the Lagrangian Particle Dispersion Model Spray, Some Case Studies. Air Pollution Modeling and Its Application XIII.

[42] Arianet Company. http://www.aria-net.it/.

[43] UNI EN Standard EN 16147:2017 Heat Pumps with Electrically Driven Compressors-Testing, Performance Rating and Requirements for Marking of Domestic Hot Water Units. http://bit.ly/2Ecra14.

[44] ARPA Piemonte. http://www.arpa.piemonte.it/.

[45] ARIA Technologies. http://www.aria.fr/.

[46] Anenberg S.C., Belova A., Brandt J., Fann N., Greco S., Guttikunda S., Heroux M.-E., Hurley F., Krzyzanowski M., Medina S. (2016). Survey of Ambient Air Pollution Health Risk Assessment Tools: Survey of Ambient Air Pollution Health Risk Assessment Tools. Risk Anal..

[47] European Commission ExternE-Externalities of Energy: Methodology 2005 Update. http://www.externe.info/externe_d7/.

[48] WHO Health Risks of Air Pollution in Europe–HRAPIE Project Recommendations for Concentration–Response Functions for Cost–Benefit Analysis of Particulate Matter, Ozone and Nitrogen Dioxide. http://bit.ly/2YNPrnS.

[49] Holland M. Cost-Benefit Analysis of Final Policy Scenarios for the EU Clean Air Package-Version 2, Corresponding to IIASA TSAP Report 11, Version 2a. https://ec.europa.eu/environment/air/pdf/TSAP%20CBA.pdf.

[50] Eurostat, Harmonized Indices of Consumer Prices (HICP)-Main Tables. http://ec.europa.eu/eurostat/web/hicp/data/main-tables.

[51] Agenzia delle Entrate Ristrutturazioni Edilizie: Le Agevolazioni Fiscali (Building Refurbishment: Tax reliefs). http://bit.ly/2PfpN8f.

[52] BOSCH DHW Heat Pumps. http://bit.ly/36RHKj5.

[53] OCHSNER Heat Pumps Catalogue. http://bit.ly/34G9zt8.

[54] DAIKIN Listino Prezzi Riscaldamento Aprile 2019 (Heating Appliances Price List-April 2019). http://bit.ly/2sIRE7Z.

[55] Eurostat Energy Prices in 2018. Household Energy Prices in the EU Increased Compared with 2017 +3.5% for Electricity and +5.7% for Gas. http://bit.ly/34dKjKB.

[56] ARPA Lombardia; Regione Lombardia; Regione Piemonte; Regione Emilia Romagna; Regione Veneto; Regione Friuli-Venezia Giulia; Regione Puglia; Provincia di Trento; Provincia di Bolzano INEMAR—INventario EMssioni ARia (Inventory of Emissions of Air Pollutants). http://www.inemar.eu/xwiki/bin/view/Inemar/WebHome.

[57] Comune di Torino 2011 Census Yearbook. http://bit.ly/36UJCb0.

